# Work-Related Stress in the Banking Sector: A Review of Incidence, Correlated Factors, and Major Consequences

**DOI:** 10.3389/fpsyg.2017.02166

**Published:** 2017-12-12

**Authors:** Gabriele Giorgi, Giulio Arcangeli, Milda Perminiene, Chiara Lorini, Antonio Ariza-Montes, Javier Fiz-Perez, Annamaria Di Fabio, Nicola Mucci

**Affiliations:** ^1^Department of Human Sciences, European University of Rome, Rome, Italy; ^2^Department of Experimental and Clinical Medicine, University of Florence, Florence, Italy; ^3^School of Psychology, University of East London, London, United Kingdom; ^4^Department of Health Sciences, University of Florence, Florence, Italy; ^5^Department of Management, Universidad Loyola Andalucía, Córdoba, Spain; ^6^Department of Business Administration, Universidad Autónoma de Chile, Santiago, Chile; ^7^Department of Education and Psychology, University of Florence, Florence, Italy

**Keywords:** work-related stress, organizational stress, mental health, banking, occupational health, occupational medicine

## Abstract

For a number of years now, banks have been going through enormous changes in organization and structure. New technology and new ways of structuring the operation have left their mark on the working conditions and daily lives of employees. Deregulation of labor markets, emerging technologies and new types of jobs have significantly reshaping working lives by continuous changes on employment and working conditions. Such a scenario has a relevant impact not only on companies' organization but also on working population's health. The banking sector is particularly well-deserved of a specific and thorough analysis, in view of the recent increase in psycho-social disorders of employees. This may be related to the major organizational changes affecting this sector and, in particular, to the restructuring processes resulting from the global economic crisis. Our aim is to assess the scale of the phenomenon and how far it relates specifically to the processes of bank organization. With this in mind, through a review of the literature, we selected the main studies dealing with work-related stress in banking, so that we could reach a better understanding of the phenomenon as it relates specifically to this set of workers. The search took place on the MEDLINE® database; in total 20 articles were chosen. There was uniform agreement among the studies that stress in the banking workplace is now at critical levels, and that it can have deleterious psychological effects on workers, and on their physical health, and that organizations, too, are affected. Most studies showed that mental health problems had increased in the banking sector, and that they were stress-related. Examples began with anxiety and depression, carried on through maladaptive behaviors, and ended in job burnout. The reviewed studies' limitations were then discussed, and possible ways forward considered.

## Introduction

In recent decades, moves to a global economy and deregulated markets have led to a series of large changes in the way financial services work and are sold, and this is particularly true of the organization and execution of work in the sector (Hassard et al., 2017; Kaur et al., 2017). There was enormous change in the way banks were organized and the effect on the working lives of employees of new technology and new structures was severe.

The credit industry is experiencing a particularly important moment due to major changes in organizations and the global economic crisis. In Italy, the main cause of reorganization of this sector lies in the large number of internal mergers and acquisitions (M&A), mainly due to strategic reasons rather than to financial failures (Pohl and Tortella, 2017). Therefore, these M&A processes not only helped to redraw the landscape of banks but also the scenario of the banking groups themselves: on the one hand the growing integration of the Italian banking system with the European one has pushed the creation of companies of such size to compete with the large groups in the rest of Europe, on the other, the reorganization has led to a remodeling through banking groups alliances, with the resulting acquisition of a dominant market position by only three large groups.

The explosion in 2008 of the economic crisis in Europe was determined by external factors that triggered a structural crisis that was undermining the economy of some countries (e.g., Italy, Greece and Portugal) already from the beginning 2000 and prevented a proper response to economic shocks, both from the effects of the 2007 international financial crisis and the sovereign debt crisis of 2011 (Dom et al., 2016). No economic sector has been immune from the effects of the crisis, especially in the most affected countries. The repercussions in the credit sector were of two types: on the one hand, the progressive reduction in saving and investment capacity of the customers, and, on the other hand, the increasingly frequent unforeseen of the global economic market. Therefore, it is reasonable to expect consequences on the psycho-physical well-being of employees (Van Hal, 2015; Frasquilho et al., 2016).

The International Labor Organization reported a number of worrying issues for workers in financial services; these included greater pressure on time, problems with ergonomics, conflicting roles, work demands that were considered excessive, difficult relationships with customers, and a rising number of cases of stress and violence (Giga and Hoel, 2003).

Such changes have had relevant effects on bank employees, not just in the workplace but also in their daily lives. In fact, banking work, in which for at least a century there have been no major changes, has been completely redesigned. This process is inserted in a context of increased competition between national and international banks, institutional changes, implementation of economic plans, and reduced inflationary rates (Silva and Navarro, 2012; Bozdo and Kripa, 2015). The basis of the new requirements and qualifications is based on three characteristic social phenomena: unemployment, precariousness of work, and intensification of the labor rhythm (Hantzaroula, 2015).

It is possible to affirm that the substantial changes that took place with the productive restructuring were in the sense of implementing strategies such as charging clients for a greater diversity of services and products, intensification of outsourcing, flexibility of work, redefinition of tasks and traditional banking activities, and transferring more and more services to the clients themselves (i.e., through home-banking) (Silva and Navarro, 2012; Blazy et al., 2014). In this new management model, bank employees have experienced a full redefinition of their tasks, becoming bank sellers (rather than bank employees), working with clients to meet the bank's targets in areas such as the sale of investment funds, bonds, and insurance policies (Jinkings, 2004; Adrian and Ashcraft, 2016). Moreover, a considerable reduction in job positions intensified the volume of work for those who remained, as well as for new employees (Silva and Navarro, 2012).

In addition to those changes that have occurred since the process of productive restructuring have affected the way of being of bank employees, they also affected the health of workers, as a result of increasing pressure, tension and stress in the bank environment (Silva and Navarro, 2012). For some authors, both the informatization processes and the profile redefinitions can endanger the well-being and health of workers (Ganesh Kumar and Deivanai Sundaram, 2014; De Cuyper and Isaksson, 2017; Manjunatha and Renukamurthy, 2017).

The National Institute for Occupational Safety and Health (NIOSH) ranked occupations for stress levels, with some of the 130 occupations found to be more stressful. What these stressful occupations had in common was that the employee had insufficient control over the work, with employees feeling that they were trapped in jobs where they were regarded as quasi-machines rather than as people. The dozen most stressful positions were managerial, administrative and supervisory roles; if we extend the 12 most stressful occupations to 28, bank tellers feature in the list (Michailidis and Georgiou, 2005).

There is much literature to support the idea of occupational stress as a disease promoter, placing workers' social and psychological health at risk and damaging their social, professional and affective lives. Poor performance at work, a high level of absenteeism and staff turnover, and violence in the workplace all follow (Godin et al., 2005; Stansfeld and Candy, 2006; Bhagat et al., 2010; Burke, 2010; Dalgaard et al., 2017).

In light of what we have discussed, we believe that there is a strong need for a thorough analysis of the increasing spread of negative health outcomes that work-related stress may assume in a very changing organizational context as the banking one. Despite the existence of a plentiful Literature on work-related stress, the study of this phenomenon in the banking sector is still limited, although it falls into the field of collective health. This review can support stakeholders and institutions agencies responsible for monitoring workplaces to framing the issue as well as to develop prevention and protection strategies. In this manuscript, our intention is to assess the scale of the phenomenon and how far it relates specifically to the processes of bank organization. In particular, through a review of the literature, we selected the main studies dealing with work-related stress in banking, so that we could reach a better understanding of the phenomenon (epidemiology of work-related stress, health problems related to work-related stress, risk factors, health outcomes as an effect of work-related stress, consequences for the organization) as it relates specifically to this set of workers.

## Materials and methods

To find recent date scientific literature on the subject of work-related stress in the banking sector, we conducted a review following the MOOSE Group's guidelines (MOOSE is the acronym for Meta-analyses Of Observational Studies in Epidemiology) (Stroup et al., 2000). The platform chosen for the literature search was MEDLINE®.

Keywords used in the search were: “bank,” “banking,” “work-related stress,” “job stress,” “organizational stress,” and “stress,” and we searched for them in the title and in the abstract of manuscripts. In order to be included, papers had to describe the results of original studies (primary studies), they had to be published in English and their full-text had to be available online (based on both the subscriptions of the University of Florence and of the European University of Rome). No temporal filter has been adopted. MEDLINE® was last accessed on 31 July 2017.

Fist, the articles were selected on the basis of title and abstract, then on the basis of the full-text. The references of the selected articles were then examined to identify other suitable studies.

The quality of the selected studies, included in the final synthesis, was assessed according to the study design: Table 1 lists three quality grades, reflecting that, while some studies will meet the quality criteria in full, some will be deficient in some way and some will fail to meet the criteria in any way. Since there is not a universal agreement on the criteria to be included in quality assessment, the criteria listed in Table 1 were chosen by the research group, according to the expertise of each component of the team. Specifically, the type of study, the type of parameters used to investigate work-related stress, and the type of data collection were considered to assess the quality of the selected study.

**Table 1:** Description of the quality criteria used to assess the quality of the studies.

**Quality categories**	**High quality**	**Moderate quality**	**Low quality**
Type of study	Longitudinal	Longitudinal	Cross-sectional
Type of parameters used	Only objective parameters with standard definition	Objective parameters with standard definition and self-perceived symptoms	Only self-perceived symptoms or no parameter assessed
Type of data collection	Data collected 3 or more times over the period taken into account	Data collected just two times over the period taken into account	Data collected in a single interview or questionnaire or not collected at all

Longitudinal studies are those that follow a cohort of workers for an extended period, permitting changes in the incidence of pathological facets of banking sector work-related stress to be assessed while taking into account observed changes in both physical and psychological working conditions. There is no doubt that such longitudinal studies, as opposed to cross-sectional, studies, would produce more accurate results. Subjective (self-perceived) parameters could lead to social desirability bias, which could result in misclassification or in bias in the interpretation of objective parameters. Moreover, studies with data collected three or more times were considered more accurate and so with higher quality.

## Results

About 600 citations (565 articles) were identified thought the search strategy. The selection by title and abstract led to the exclusion of 530 studies not related specifically to the topic or not in agreement with the inclusion criteria (not primary studies, published in any language different from English, full text not available). The selection by full text led to the exclusion of 20 papers. By consulting the reference lists of the selected articles, five papers were identified. So, the procedure set out above, led to the selection of 20 articles, of which five were European (Spain, Cyprus, the Netherlands, Italy, and Iceland), nine were Asian (India, Pakistan, China and Malaysia), four came from Brazil, and two were African (South Africa, Nigeria).

Table 2 summarizes results drawn from the studies concerning work-related stress and its effects, with particular reference to the banking sector.

**Table 2:** Summary of the studies included in the review.

**Authors**	**Year**	**Journal**	**Results**
Seegers and van Elderen	1996	European Journal of Psychological Assessment	Subjective stressors contributed to work-related stress; lack of knowledge and lack of responsibility were related to an increased perception of meaninglessness of work and job future uncertainty; meaninglessness of work was related to job dissatisfaction; cognitive anxiety was related to psychological complaints and psychological complaints to health complaints. Social support had a compensating effect, tending to reduce the negative effect of stressors.
Mocci, Serra and Corrias	2001	Occup Environ Med	Social support, group conflict, self-esteem, work satisfaction and underuse of skills were found to be predictors of visual complaints; social support played a part also as a moderating factor in the stress and strain model; this model accounted for 30% of the variance.
Michailidis and Georgiou	2005	Work	Employees' educational levels affected the degree of stress they experienced in various ways; the degree to which some employees tended to bring work-related problems home depended on their educational background, the strength of the employees' family support, and the amount of time available for them to relax. The drinking habits were found to play a significant role in determining the levels of occupational stress.
Silva and Barreto	2010	BMC Public Health	Adverse working conditions assessed using demand control (Karasek, 1979) and effort-reward imbalance (ERI) model (Siegrist, 1996) were statistically associated with the presence of minor psychiatric disorders. Compared to workers exposed to low-demand and high-control activities, the prevalence of MPD more than doubled among those in maximum demand and minimum control conditions.
Mughal et al.	2010	J For Global Business Advancement	Importance of stress factor toward work-life balance. Stressors are directly proportional to work-life balance. The organizational source of stress (task demands, role demands, organizational structure, organizational leadership, interpersonal demand and job security) has a valid impact on work-life balance.
Ahmad and Singh	2011	International Journal of Management and Strategy	A few stressors of occupational stress scale have been found to have a causative influence on banking sector employees' perceived reactions toward Organizational Change: responsibility for persons, intrinsic impoverishment, low status and unprofitability. Among biographical variables, only “experience in the present position” was a predictor of banking sector employees' reactions.
Makhbul et al.	2011	Australian Journal of Basic and Applied Sciences	A large percentage of the changes in stress outcomes in the workplace were due to its relationship with body postures and health factors. The body posture had a noticeable effect and was significantly related to stress outcomes at the workplace.
Mutsvunguma and Gwandure	2011	Psychology, Health and Medicine	Significant differences between the psychological well-being of bank employees who handled cash and those who did not handle cash. They differed in terms of work stress, emotional exhaustion, depersonalization and overall burnout.
Silva and Barreto	2012	Journal of Occupational Health	Relationship between exposure to adverse psychosocial work environment and poor self-rated health. This effect was seen in both demand-control and ERI models.
Fernandes et al.	2012	International Journal of Behavioural and Healthcare Research	Authors extracted specific dimensions that could promote the reduction of stress. Human support was the factor that most reduced total stress, followed by relaxed health practices and by vigorous health practices.
Snorradóttir et al.	2013	American Journal of Industrial Medicine	The risk of psychological distress depends on the extent of change experienced and the level of entanglement in the process. Environment factors such as high job demand and low job control played a part in perceived psychological distress, but only to a limited degree. The negative effects of the psychological distress could be partly attenuated by the empowering leadership.
Devi and Sharma	2013	IIMB Management Review	Frontline bank employees differed significantly on the basis of their experience of role stressors and merited categorization into distinct segments: “overloaded employees,” “unclear employees” and “underutilized employees.” The profiles of the frontline bank employees falling in the above distinct segments were also found to be significantly different.
Oginni et al.	2013	International Journal of Business and Management Invention	Job security is the greatest source of job stress to Nigerian bankers, followed by work materials made available by the management of the institutions; next came organizational policies that guided the activities and decisions of employees. After this was work pressure, which can be said to be a follow-up to the organizational policies.
Amigo et al.	2014	Psicothema	High degree of Burnout Syndrome amongst employees of Spanish Savings Banks. The factor for which the greatest number of workers showed a high risk of BS was emotional exhaustion. Working in branch offices implied a higher risk of suffering from burnout than working in central services.
Preshita and Pramod	2014	International Journal of Applied Business and Economic Research	Both private and public sectors experienced moderate to high levels of stress: role stagnation emerged as the most potent role stressor in both sectors, followed by inter-role distance and role erosion. Employees of private sector banks had higher total ORS scores compared to public sector banks.
Imam et al.	2014	Middle-East Journal of Scientific Research	Stress played a vital part of partial mediator in intensifying and strengthening the impact of gender discrimination-glass ceiling on job satisfaction and employee motivation.
Petarli et al.	2015	Ciência and Saúde Coletiva	The important role of social support, considered the most well-known situational variable against occupational stress (Bakker and Demerouti, 2007), was evident in the study. Low social support increased the likelihood of belonging to the “high distress” quadrant.
Valente et al.	2015	Occupational Medicine	Having a job characterized as high strain, low social support, high effort/low reward and high over-commitment was strongly associated with both major and other depressive symptoms. Strong association between low social support and depressive symptoms.
Li et al.	2015	International Journal of Environmental Research and Public Health	The average scores of the three dimensions of job burnout in this sample were higher than in five occupational groups from three nations (Finland, Sweden and the Netherlands) (Schutte et al., 2000). The main contribution of the study is to highlight that occupational stress may affect the risk of job burnout in bank employees via a mediating mechanism of PsyCap.
Kan and Yu	2016	International Journal of Environmental Research and Public Health	Chinese bank employees suffer from high levels of depressive symptoms. A significantly negative association of PsyCap with depressive symptoms among Chinese bank employees. Occupational stressors from ERI (extrinsic effort and reward) were significantly associated with PsyCap. PsyCap partially mediated the associations of extrinsic effort and reward with depressive symptoms.

Considering the study design, no studies are classified in the first, completely satisfactory, quality category (Table 1), because not one of the examined studies met every quality criterion. The most obvious reasons for this are that there are no longitudinal studies into how banking sector work-related stress affects employees, and that the studies al rely on respondents self-assessing and self-reporting, so that there is a risk of social desirability bias. In fact, all publications were assigned in the low quality category.

The oldest study included in this review was published in 1996; the other researches are quite recent, and 17 (85%) were published in the last 10 years.

Five of the articles examined ways in which stress at work might give rise to various negative mental health and physical results. These included anxiety and depression, together with such maladaptive behaviors such as smoking and drinking (Silva and Barreto, 2010, 2012; Snorradóttir et al., 2013; Petarli et al., 2015; Valente et al., 2015). Another five investigated how stress at work can give rise to such specific conditions as workplace discrimination, work-family conflict, (lack of) job satisfaction and employee motivation, high staff turnover and work-life imbalance in employees (Seegers and van Elderen, 1996; Mughal et al., 2010; Oginni et al., 2013; Imam et al., 2014; Kan and Yu, 2016). A further three looked for an occupational stress/job burnout nexus (Mutsvunguma and Gwandure, 2011; Amigo et al., 2014; Li et al., 2015). In two cases, the focus was those factors in the banking sector that could make or increase stress levels in particular roles (Fernandes et al., 2012; Devi and Sharma, 2013). Two more were concerned with the role played in development of specific physical symptoms by organizational stress in the development of specific physical symptoms (Mocci et al., 2001; Makhbul et al., 2011); two others looked at factors contributing to work-related stress in banking sector employees (Michailidis and Georgiou, 2005; Ahmad and Singh, 2011); and the last looked for differences between male and female respondents and between those employed by private and by public sector banks in the kind of stress and the degree of intensity (Preshita and Pramod, 2014).

The majority of papers analyzed for our review dealt with the effects of work-related stress in the banking sector.

Seegers and van Elderen (1996) investigated how stressors related to work affected the physical and psychological well-being in a large Dutch banking organization, and what levels of absenteeism they gave rise to. Invitations to complete a questionnaire were sent to five hundred bank directors, of whom 376 (75.2%) responded by completing the forms. The basis of the questionnaire was a Dutch version of a questionnaire developed by the Institute for Social Research at the University of Michigan (Vragenlijst voor Organizatie Stress (VOS-D) for the measurement of organizational stress (Bergers et al., 1986) with the addition of objective stressors (age, years of directorship, staff size). Also added were two subscales drawn from the Self-Expression and Self-Control Questionnaire (Zelfexpressie en Controle Vragenlijst, ZECV) (Maes et al., 1987) in order to measure the degree to which anger was internalized or externalized, and questions intended to assess the degree of social support. Stressor variables included:

- Workload;
- Role ambiguity, role conflict and lack of responsibility; *and*
- Lack of knowledge.

Work-related stressors included two scales:

- Meaninglessness of work; *and*
- Uncertainty as to job future.

There was a division of work-related psychological strains into job dissatisfaction and cognitive anxiety, in addition to which were also considered:

- Psychological and health complaints; *and*
- Absenteeism (measured by self-reporting of days' absence in the past year.

The conclusion was that subjective stressors did contribute to work-related stress, while lack of knowledge and lack of responsibility expressed themselves in increased feelings that work was meaningless and the future uncertain. Meaninglessness of work and job dissatisfaction were connected with each other; so were psychological complaints to cognitive anxiety and health complaints; while a person's perceived psychological well-being was related to work-related strains.

Social support was confirmed as compensating for stress in that it tended to lessen negative effects caused by stressors and so led to reduced stress reactions. The study's limitations centered on the fact that the data came from a cross-sectional study and were for the most part self-reported and retrospective.

Mocci et al. (2001) set out to examine how far psychological stressors contribute to asthenopeic complaints (asthenopia is visual discomfort) as well as the extent to which social support could mitigate the effect of job stressors. To this end, it looked at the influence of such stressors as task, individual characteristics and social environment on asthenopia in computer users. Ophthalmological examination allowed the selection from a total of 385 Italian bank workers of 212 who did not have organic visual weaknesses, and who shared both work environment and job duties to create the study group. This group was given three questionnaires:

- the NIOSH job stress questionnaire (Hurrell and McLaney, 1988; Mocci, 1994);
- a questionnaire designed to identify subjective discomfort springing from or caused by the workplace's environment and lighting conditions; *and*
- a questionnaire on whether oculo-visual disturbances were experienced.

Results showed the following (or their lack) predictors of visual complaints:

- Social support;
- Self-esteem;
- Work satisfaction;
- Group conflict; *and*
- The underuse of skills

Social support could also act to mitigate the stress and strain model which was responsible for 30% of the variance. Although they could have a significant correlation with asthenopia, subjective environmental factors did not show up strongly as predictors of the symptoms. The authors' conclusion was that complaints about visual health by VDT workers probably reflect in part a psychological discomfort attributable to conditions of work. Discussing the limitations of their work, the researchers observed that they had evaluated environmental discomfort on the basis of self-reporting only, that the same caveat applied to occupational stressors and visual discomfort, and that using self-reports of both job stressors and strains made for a greater likelihood of conceptual overlap.

Michailidis and Georgiou (2005) studied occupational stress as it affects Cyprus's banking sector. The study improved understanding of factors contributing to occupational stress as employees in this industry feel it. The authors evaluated the extent of any correlation between employees' educational levels, relaxation patterns, and smoking and drinking habits with their perceptions of their level of occupational stress. Subjects, drawn from both genders and a variety of educational backgrounds, completed the Occupational Stress Indicator (OSI) (Cooper et al., 1988) with a view to establishing the degree to which occupational stress affects varying groups. 80 full-time employees of different banks in Nicosia, the Cypriot capital, were chosen at random to receive the questionnaires were distributed to 80 randomly selected. There were significant differences in the results between employees educated to higher degree level and those without formal qualifications. It was those educated to higher degree level who were most affected by the impact of the home/work interface; they showed a tendency to take work problems and demands home with them, and to appear to be following career objectives to the detriment of their home life. They were also the ones most likely to feel the brunt of such factors as: variety of work; delegation; favoritism; and conflicting tasks. The study also showed that, faced with high levels of these issues, these were the employees most likely to reduce the amount of time they had for themselves, thus losing relaxation time. Finally, employees in the habit of drinking alcohol were shown to have difficulty seeking advice from their supervisors and to be reluctant to look for social support. This research suggests that an employee with more educational qualifications will be more likely to act in a more detached and unemotional way and be more objective in order to reduce the impact of stress.

Silva and Barreto (2010) took as their study's starting point Nakao's work (2010) on work-related stress and how it connects to a variety of negative outcomes in both physical and mental health, including depression and anxiety, together with such maladaptive behaviors as smoking and drinking. The aim of the study was to estimate how prevalent minor psychiatric disorders (MPD) were in the employees of a large bank in Brazil, and to investigate how far these could be related to adverse psychosocial working conditions. Their methodology embraced demand control (Karasek, 1979) and the effort-reward imbalance (ERI) model (Siegrist, 1996). Authors also examined the degree to which social support or the presence of over-commitment could modify these associations, as suggested by, respectively, the JCQ and ERI models, respectively. A version of the General Health Questionnaire containing 12 questions (GHQ-12) adapted by Mari and Williams, tested for the presence of MPD (Mari and Williams, 1985), while two tools were used to assess psychosocial factors at work: the JCQ reduced version that Araújo had adapted to Portuguese (2003), and the version of the ERI scale that Silva and Barreto had adapted to Portuguese by (2010). On the basis of assumptions in Karasek's model, the median value was used to dichotomize variables, which were combined in four distinct categories. The highest exposure group contained those workers faced with a combination of low control and high demand. Intermediate groups were those for active job (high control and high demand) and passive job (low demand and low control). Eighty-eight percent of the 2,337 eligible workers took part in the study, and results suggested a statistical association with MPD for adverse working conditions in both scales. MPD prevalence more than doubled among those facing maximum demand while exercising minimum control. ERI produced similar results, with MPD prevalence varying from 33% (low-effort and high-reward employees) to 70% for high effort/low reward staff. Absence of social support at work and over-commitment both also showed a statistical association with MPD. Limitations of the population and design, the healthy worker effect and data collection through self-response were present in the study and. the study's cross-sectional nature prevents inferences from being drawn on the causal nature of associations thrown up between common mental disorders and stress at work. Finally, as MPD is a major cause of temporary leave and invalidity pensions among bank workers in the country (Silva et al., 2007), the authors commented on the possibility that there was no participation in this work by individuals with severe mental disorders, so that the prevalence of MPD in the study population would be underestimated. The authors went on to suggest that new studies of bank workers be developed, and especially those of a longitudinal design, in an attempt to understand the mechanism of these associations. Ahmad and Singh (2011) investigated how occupational stress and a number of biographical variables in a sample of 350 bank employees randomly drawn from various Indian banks (age, experience in present position, total experience, salary and number of dependents) affected employee reactions to organizational change (OC). A scale developed by Rahman (1993) was used to measure organizational change, while the Occupational Stress Index−46 items covering 12 aspects of occupational stress (role ambiguity, role overload, role conflict, powerlessness, under-participation, unreasonable group and political pressures, responsibility for persons, poor peer-relations, low status, intrinsic impoverishment, strenuous working conditions, and unprofitability) was used to gauge occupational stress. The authors found a causative influence on employees' perceived reactions to OC in certain stressors from the occupational stress scale; these were: responsibility for persons, intrinsic impoverishment, low status and unprofitability. The only biographical variable to predict banking sector employees' reactions to organizational change was “experience in the present position.”

Silva and Barreto (2012) examined how far poor self-rated health of Brazilian employees in financial services could be connected to adverse psychosocial working conditions. In a cross-sectional study, a random sample of 2,054 bank employees (49.7% male and 50.3% female) answered a questionnaire containing questions about five areas of interest: socio-demographic, health, psychosocial, behavioral and work-related factors. Among the latter were: length of service in the company; current job; and psychosocial nature of the job. The demand-control model (Karasek, 1979) and effort reward imbalance (ERI) model were used to assess independent associations with the outcome (Siegrist, 1996). Employees with a high demand/low control work combination appeared in the group that had most exposure to stressful conditions. High demand/high control employees were placed in the intermediate exposure group, together with low control/ low demand employees, while employees working under low demand and high control provided the reference category for statistical analysis, since they were seen as having no exposure to stress. For the ERI model, a work-related stress index was constructed using cutoffs derived from the distribution's tertiles (Jonge et al., 2000). Of these, the highest was the most exposed group; the intermediate exposure group was second, and in the first tertile were the unstressed reference category. To find the dependent variable for the analysis, a single question was used: “In general, compared to people of your age, would you say your health is (…)?” Five responses were possible: very poor; poor; good, very good; or excellent). A statistical association with poor self-rated health was found with advancing years; lack of physical activity; chronic diseases; problems sleeping; regular medication use; and having been employed by the company for between 6 and 14 years. Exposure to high strain work and lack of social support at work also showed a statistical association with poor self-rated health. These results added further support to the idea of a relationship between poor self-rated health and exposure to an adverse psychosocial work environment, an effect seen in both demand-control and ERI models. In the first of those models, the association was stronger both among high strain/low control workers and employees lacking social support at work, while in the ERI model, the likelihood of poor self- rated health increased for high effort-reward imbalance and a high level of commitment at work.

The subject of study for Snorradóttir et al. (2013) was psychological distress caused to employees by rapid and unpredictable changes when the organizations they worked for underwent financial collapse. What they examined was the level of psychological distress among those employees who survived the crash, and the impact on them of involvement in the twin processes of downsizing and restructuring. The study was cross-sectional in design; data were collected from all the headquarters and branch‘ employees in all of the three Icelandic banks that collapsed during the first week of October 2008. The basis of the questionnaire used was the General Nordic Questionnaire for psychological and social factors at work (short version) (QPS-Nordic 34þ), with some questions from the long version added (Dallner et al., 2000), together with questions culled from the Copenhagen Psychosocial Questionnaire (COPSOQ) (Kristensen et al., 2005) and from questionnaires that had previously been administered by the Icelandic Public Health Institute (Jónsson and Gudlaugsson, 2010) and the Administration of Occupational Safety and Health (AOSH) (Rafnsdottir and Gudmundsdottir, 2004; Sveinsdóttir and Gunnarsdóottir, 2008). Completed questionnaires were returned by a total of 1,880 employees. The scale used to measure psychological distress was one previously used by AOSH (Sveinsdóttir and Gunnarsdóottir, 2008) including five items indicating symptoms of depression, anxiety, being overly concerned, sleep disturbances, and feeling exhausted. What was demonstrated is that workers who were more entangled in the downsizing or restructuring processes were more distressed than those who were not. Higher distress levels were seen in bank employees working in downsized departments, transferred to a different department, or having taken a salary reduction and the likelihood of psychological distress varied according to how much change had been experienced and how deeply involved in the process the person was. The researchers' conclusion was that factors relating to psychosocial work environment played a part, but only a limited part, in perceived psychological distress for those involved in the organizational change processes in the early aftermath of the collapse of the banks. Also, results indicated that psychological distress in employees could be reduced during difficult economic times if management prioritized employee well-being, informing and encouraging employee participation in decision-making (Arnetz, 2005; Svensen et al., 2007), and that distress was reduced in such employees when friends and family provided social support. Coworker support or support from supervisors, however, was not so effective. This study gains strength from having been, uniquely, nationwide with every employee of a collapsed major bank in one country being involved. As with the other studies, the central weakness was its cross-sectional nature and use of self-reported data (Kompier, 2002).

In the first large-scale study carried out in the Spanish banking sector, Amigo et al. (2014) investigated how prevalent employee burnout syndrome (BS) is. One aim of the study was to differentiate between commercial branch office staff who dealt with the general public, and central services employees. Participants in the study came from all the Spanish Savings Banks and totaled 1,341, of whom 883 were men and 458 women. 1,130 worked in branch offices and had direct contact with the clients; the number of central services workers was 211. The Maslach Burnout Inventory-General Survey (MBI-GS) (Schaufeli et al., 1996), adapted for the Spanish environment by Salanova et al. (2000), was used. While the study showed high BS rates among employees of Spanish savings banks, and 63.16% of participants showed high levels of risk, significant differences emerged between the two groups in all three dimensions of BS: emotional exhaustion (*p* < 0.001); cynicism (*p* < 0.001); and professional efficacy (*p* < 0.001). Branch office workers returned higher scores for emotional exhaustion and cynicism and lower scores for professional efficacy. This high incidence of BS was higher even than that shown by other studies where the professions involved are associated with BS (Hernández et al., 2006; Longas et al., 2012) and the high rate of BS shown becomes even more relevant when one takes into account that the criteria used to establish risk was very restrictive; only workers who posted high scores in a minimum of two factors of the MBI-GS were counted. Emotional exhaustion was the factor involving the greatest number of workers showing high BS risk of BS. The difference between groups may be interpreted as arising from the likelihood that branch office workers will have daily contact with people whose economic problems are severe, and that they had to deal with these problems. What's more, branch workers were required to sell financial products with a problematic past illustrating the very competitive practices among financial institutions. All these things led to a certain loss of control for branch office employees and might be seen as explaining their feelings of less professional efficacy, greater emotional exhaustion and greater cynicism (Alarcon, 2011; Lee et al., 2011). A branch office job carries more risk of burnout than one in central services. The significant difference was contact with the public, which branch office staff have and central services personnel do not. The suggestion from these results is that burnout has more to do with daily interpersonal stress at work, exacerbated by the sector's commercial strategies of recent years, than to the possibility of being let go or asked to take a cut in salary.

The incidence of occupational stress in Brazilian bank employees, and its association with socioeconomic, demographic, and labor characteristics, was studied by Petarli et al. (2015). This was a cross-sectional study of 521 people aged between 20 and 64 and drawn from both sexes who worked for a banking network in the state of Espirito Santo. Data collection took place between August 2008 and August 2009. The short version of the Job Stress Scale (Alves et al., 2004), adapted for the Brazilian market and developed according to the demand-control model, was used (Karasek et al., 1998). The demand-control model has four quadrants: high distress (high-demand/low-control jobs), passive job (low-demand/low-control jobs), active job (high-demand/high-control jobs), and low distress (low-demand/high-control jobs). The study placed the biggest number of bank employees (*n* = 179, 34.5%) in the “passive” quadrant; the drawback to passive jobs lies in their combination of low psychological distress and low control, possibly leading to a gradual decrease in learning and skill development. The greatest risk of occupational stress lay with the quadrant that had the lowest number of employees. These results were not those expected, given the factors in today's banking activities—demanding targets, fierce competition, fewer available jobs, a constant demand to increase qualifications, intensified and overloaded tasks, and increased control and pressure on workers—that could increase the occupational stress risk. The control-demand model concentrates on processes of workforce organization to assess the risk of stress (Alves et al., 2004) and takes no account of other important facets of development and perception of occupational stress, so that a broader evaluation of stress levels in the employees studied might have been possible. Social support, regarded as the most well-known situational variable for occupational stress analysis (Bakker and Demerouti, 2007), was shown in the study to play an important role: Where there was low social support, there was a greater likelihood of being in the “high distress” quadrant. This variable's efficacy in reducing predicted occupational stress was borne out by results from other studies (Leong et al., 1996; Urbanetto et al., 2011). Finally, those variables making up quadrants associated with increased occupational stress risks were:

- Low education levels;
- Working in bank agencies;
- More than 5 years' employment at the bank;
- Six hour daily work shifts; and *especially*
- Low levels of social support.

Conversely, the following correlated with a lower risk of occupational stress than being single:

- Being married;
- Living with a partner; *and*
- Being separated, divorced, or widowed.

A sample of 1,046 employees in financial services in northern Brazil allowed Valente et al. (2015) to look for correlations between:

- Psychosocial conditions in banking work, as assessed by the Job Demand-Control-Support model (Karasek et al., 1998) and Effort-Reward Imbalance (ERI) at work model (Siegrist et al., 2004); *and*
- Major depressive symptoms and other forms of depressive symptoms.

This cross-sectional study was conducted between 2012 and 2013, with data obtained through a self-administered questionnaire that combined the Patient Health Questionnaire-9 (PHQ-9) (Fraguas et al., 2006), the Demand–Control–Support Questionnaire (DCSQ), adapted to Brazilian Portuguese (Alves et al., 2004), and the Brazilian version of the ERI-Questionnaire (ERI-Q) (Chor et al., 2008) to assess:

- Work, social and demographic issues;
- Depressive symptoms; and
- Psychosocial aspects of the working environment.

Thirty-two percent of bank employees were found to have depressive symptoms which would justify a diagnosis of clinical depression. There was a strong correlation between, on the one hand, depressive symptoms both major and otherwise to be characterized and, on the other, being in work that could be seen as high in strain, low in social support, combining high effort with low reward, and a high level of over-commitment. The authors also saw a strong correlation between low social support levels and symptoms of depression, which confirmed previous findings (Wang et al., 2011; Yu et al., 2013); in sum, employees whose working environment combined high strain with low social support levels were more likely to have health problems than were with those whose social support levels were high. The conclusion was that banking sector work demanded constant skill updating to stay abreast of new work organization forms. Employees, and especially older employees, could find this threatening and experience additional stress as a result (Giga and Hoel, 2003). A private banking sector job might also bring with it the possibility of instability of employment, with downsizing resulting in pressure overload and stress a major employee concern.

According to Li et al. (2015), there had been no examination of the role of Psychological Capital (PsyCap) in mediating between occupational stress and job burnout in bank employees. Their study of a Chinese population filled this gap by examining potential mediation of PsyCap on occupational stress leading to job burnout. Their aims were:

- To determine what association, if any, existed between occupational stress and job burnout;
- To appraise the association between PsyCap and job burnout; and
- To explore whatever efficacy PsyCap might have in mediating the occupational stress/job burnout connection in the case of Chinese bank employees.
- This study was to be carried out separately for male and female employees

The study was cross-sectional survey by nature and carried out in northeast China from June to August 2013. Self-administered questionnaires were returned by 1,239 employees. The Chinese version of the ERI questionnaire was used to assess occupational stress, with a claim to examine failed reciprocity in cases where substantial efforts are met with low rewards (Yang and Li, 2004). PsyCap was measured using the Chinese version of the 24-item Psychological Capital Questionnaire (PCQ) (Luthans et al., 2007), while the Chinese version of the Maslach Burnout Inventory-General Survey (MBI-GS) was used to measure job burnout. Average scores of all three job burnout dimensions exceeded those in five other occupational groups (managers, clerks, foremen, technical professionals, and blue-collar workers) in Finland, Sweden and the Netherlands (Schutte et al., 2000). There was no difference in correlation of occupational stress with the three occupational stress dimensions between male and female bank employees. A positive relationship was found between extrinsic effort and over-commitment with, respectively, emotional exhaustion and depersonalization. It follows that tiredness is less likely in bank employees whose PsyCap is adequate and that their burnout symptoms were likely to show improvement. The implication was that PsyCap might have a strong effect on job burnout and that it could prove an effective resource in combating job burnout among Chinese bank employees. There was, though, a gender difference in how PsyCap mediates the association between occupational stress and job burnout: male bank employees found that PsyCap mediated the effects of extrinsic effort and reward on emotional exhaustion and depersonalization, while female bank employees found that PsyCap partially mediated effects of extrinsic effort, reward and over-commitment on the same two job burnout dimensions. The authors said that the study's main contribution was highlighting occupational stress's possible role in affecting bank employees' job burnout risk through PsyCap as mediator So that, as well as reducing the amount of excessive effort and over- commitment and increasing rewards, bank administrators could use PsyCap more to reduce job burnout among their employees, and especially their female employees.

Makhbul et al. (2011) investigated which factor in the ergonomic workstation variables in the Banking Supervision Department in ABC Bank in Malaysia had the most influence on stress levels. Thirty-one employees of department took part in this study. Several questionnaires having to do with ergonomic workstation factors and resulting stress at work inherited from past research contributed to developing questionnaires; questions included:

- Human and environment variables in the matter of ergonomic workstations; and
- Lists of:
  - physiological (somatic complaints),
  - psychological (job dissatisfaction complaints),
  - behavioral (intention to quit) elements.

47.2% of changes in workplace stress levels were shown to be due to alterations in posture and other health matters. Factors relating to ergonomic workstations included the effect of posture which correlated significantly with workplace stress levels. Health was shown on analysis to have a stronger relationship than any other factor with workplace stress due to the hours of input work demanded in the department. Dissatisfaction with the job, somatic illnesses and a decision to leave were all fueled by postural weaknesses due to ergonomic considerations related to the workstation and health factors.

Fernandes et al. (2012) conducted a study to measure how organizational, environmental and health matters affected organizational role stress (ORS). Data was collected in a survey of 483 respondents in both public and private banking sectors in Goa, India. ORS was measured using a technique of Pareek (1983), which indexed the stress individuals perceived in their role, split across 10 categories: The extent to which the role is ambiguous; what is expected of the role; inadequacy of available resource; stress arising from the content of the role; erosion of the role; isolation of the role; perceived distance between self and role; stress due to the magnitude of the role; stagnation of the role; perceived distance between roles; role overload; and a sense that the personal assigned to the role is inadequate to perform it. In looking for the categories that could best encourage stress reduction, the study selected human support as the most effective. Second came health regimens that encouraged relaxation like meditation and yoga, together with more vigorous health pursuits like jogging and playing games. The study found a negative correlation between role definition stress and a combination of meditation, human and physical support, and yoga. When it came to reducing stress due to the magnitude of the role, human support again scored the highest correlation, followed by the more vigorous activities. The authors defined the study's limitations as difficulty in generalizing the findings to fit a wider range of bankers, which would require responses from a more diverse sample.

Preshita and Pramod (2014) set out to understand what contributed to occupational stress in Indian private and public sector banks. With 230 respondents collected by convenience and random sampling through the ORS scale (Pareek, 1983), they looked for differences in the kinds of stress felt and the strength with which it was experienced, looking also for male/female and public/private sector differences The study found that stress was experienced, at levels ranging from moderate to high, in both sectors, with stagnating roles being the most powerful stressor across either sector; distance between roles and erosion of roles came next. ORS scores were higher in private sector employees. According to the authors, stagnation of roles is inherent in the job–banking contains many monotonous jobs and employees who have repeated the same task again and again over a long period without career growth opportunities and with no likelihood of future change are likely to feel that their capacities are not used to the full, that there are no learning opportunities, and to be stressed by the perception that their role is stagnating.

The study by Imam et al. (2014) looked at ways in which job stress can mediate between discrimination in the workplace and such outcomes as motivation and job satisfaction in banking in Pakistan. Types of discrimination considered were mainly to do with gender distinctions. From a random sample of three banks, the study found that in corporate banking the dependent variable had a marked predictive validity, with job stress acting in partial mediation between the two conditions, showing the role played by stress in increasing the extent to which discrimination against one gender affects motivation and job satisfaction.

The study by Mughal et al. (2010) also looked at Pakistani banks and their current issues regarding stress related to work. In this study, the subject was how stress affects work-life balance. It follows Robbins (2003) in seeing the environment, the organization and the individual as sources of stress and the results thereof (Gunkel et al., 2007). For this research only organization was used as a source of stress; job security or insecurity was added, together with others related to environment. 200 employees in middle and lower executive posts in banks responded. Six elements, or stressors, subordinate to organization, were chosen as independent variables and questions focused on each of these, an additional goal being to establish how important each was. The research uncovered the contribution to employee work/life balance of each aspect of the stressor and the scale of that relationship. Six factors (demands of the task, demands of the role, interpersonal demands, the structure of the organization, quality of leadership and security in the job) brought about a change of 63.2% change in work-life balance. The relationship between work/life balance and employee performance was also clarified. Work/life balance was strongly impacted by issues around security in the job. The conclusion was that those factors listed earlier in this paragraph caused stress and interfered with both the working and the personal lives of employees.

Occupational stress, conflicting demands of family and work, and their effect on symptoms of depression were studied by Kan and Yu (2016), who also examined what effect PsyCap had on those same symptoms and whether PsyCap could mediate the impact on symptoms of depression of occupational stress and conflicting demands of family and work. The sample was drawn from employees of Chinese banks for a cross-sectional survey from May to June 2013. With informed consent confirmed in writing, self-administered questionnaires were distributed directly and 1,239 sets of complete responses were returned. The levels of symptoms of depression and stress at work were measured by, respectively, a Chinese version of CES-D, the Depression Scale of the Center for Epidemiologic (Radloff, 1977) and the ERI scale, Chinese version (Li et al., 2006). To measure levels of conflict between family and work, the study used a Chinese version of the WIF scale (which assesses how far demands of work interfere with obligations to family) (Netemeyer et al., 1996) and PsyCap levels were measured using a Chinese version of the Psychological Capital Questionnaire (PCQ) (Luthans et al., 2007). Overall, the results may be said to have shown high levels of symptoms of depression among Chinese bank employees and the study called for urgent efforts to tackle these symptoms. The correlation between PsyCap and symptoms of depression was shown to be substantially negative. Also, extrinsic effort and reward as ERI occupational stressors showed significant association with PsyCap, leading researchers to examine the possible use of PsyCap to predict symptoms of depression. PsyCap may, in fact, be seen as partially mediating these associations, and PsyCap could act as a resource for alleviating symptoms of depression in employees of Chinese banks, reducing negative effects on mental health of occupational stress. Bank employees experiencing greater extrinsic effort or lower reward levels would be likely to have lower PsyCap levels, raising the risk and seriousness of symptoms of depression.

In South African banks, Mutsvunguma and Gwandure (2011) compared the psychological well-being of employees who handle cash with those who don't in a cross-sectional study of 50 respondents. Levels of work stress, burnout and satisfaction with life were measured using, respectively, the Job Stress Survey Scale, the Maslach Burnout Inventory and the Congruity Life Satisfaction Scale. There were significant differences between the two groups in the areas of depersonalization, emotional exhaustion, work stress, and overall burnout. Inner-city Johannesburg employees handling cash experienced work-related stress, possibly connected with the very common Johannesburg incidence of unpredictable violence to which bank employees in Johannesburg are subject (Carter, 2010; Harrison and Kinner, 2010). This was a limited study, involving a small sample and not being generalizable to different settings.

Devi and Sharma (2013) used a random sample of 501 workers to examine how stressors affected frontline bank employees in India, role stressors being events demands and constraints creating role stress by affecting an individual's role fulfillment (Beehr and Glazer, 2005). Role stress was measured using indicators from a 50-item five-point Likert type “organizational role stress” scale developed by Pareek (1993) and assessed by 15 experts identifying scale items that did not truly measure role stress in the frontline bank employee context. The experts' suggestions were incorporated to produce a final role stress scale of 22 items. Frontline bank employees were shown to differ enough in their experience of role stressors to be categorized into segments: “overloaded employees,” “unclear employees,” and “underutilized employees.” The profiles of the employees who fell into these segments were also significantly different. Overloaded employees experienced workloads heavier than they expected (high role excess). The works could also be monotonous and routine (high role fortification), leading to a compromise between work quality and time for family, friends and other personal interests (high role invasiveness). Unclear employees lacked enough knowledge to meet the responsibilities of the role (high role divergence) and/or experienced ambiguous work situations (high role indistinctness). Underutilized employees saw few growth opportunities for themselves (high role augmentation), and/or were unsure they had the necessary skills and knowledge (high self-diminution) which led to isolation at work (high resource shortage). The study made clear the need for customized approaches to role stress management. Present in the study were the following limitations: cross-sectional data; self-reported measures; and a context-specific scale that could not be generalized for other contexts without validity and reliability testing.

Oginni et al. (2013) investigated how job stress affected staff turnover in the Nigerian banking sector. This study identified job stress variables including personal problems, organizational and institutional policies, work materials, work pressure and environment, and job security. 533 respondents took part and job security was revealed as the biggest source of job stress to Nigerian bankers, with work materials second and organizational policies guiding employee activities and decisions third. Then came work pressure, which may be regarded as a follow-up the organizational policies. Work environment, institutional policies and personal problems were also important sources of stress for bankers. Job stress variables already listed had a significant effect on staff turnover. The conclusion was that job stress variables were important indicators and should be connected to staff turnover for better workforce productivity and stability (Abang et al., 2009).

## Discussion

Most of the selected studies were published in the last 10 years: the work-related stress in the banking sector is a rather recent issue, and this could be the result of the enormous changes in organization and structure that have been occurred in the last decade.

**Figure 1:**
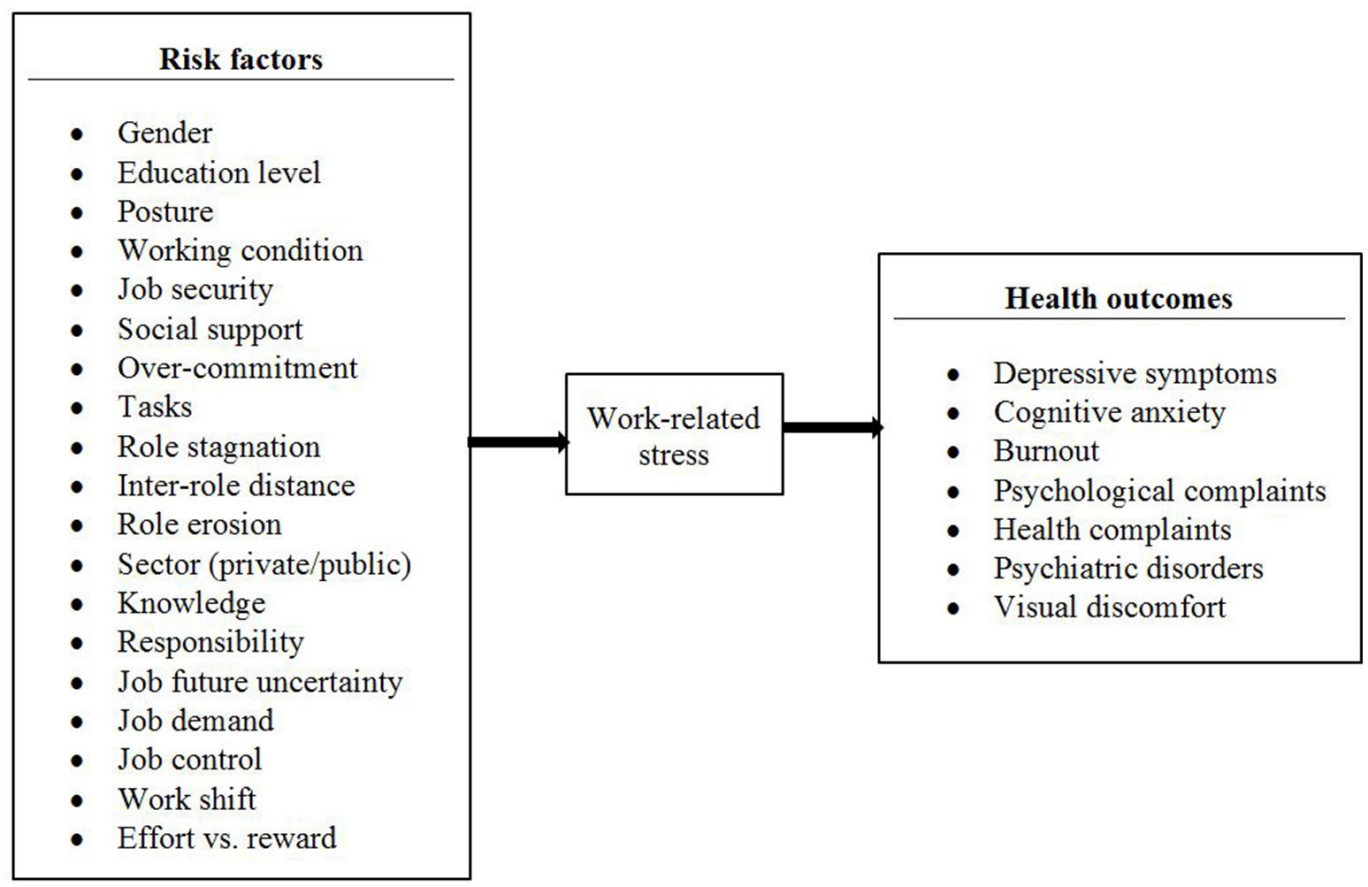
Risk factors and health outcomes of work-related stress in the bank sectors: synthesis form the studies included in this review.

Five studies were conducted in Europe, nine in Asia, four in America and two in Africa. Many differences occurred between countries and ethnicities as regards to cultural characteristics and the approach to work-related stress, that result in different risk perception and evaluation (Zoni and Lucchini, 2012; Capasso et al., 2016) and could limit the generalizability of the results across countries. Despite that, this review provides an overview on risk factors and health outcomes related to work-related stress in the banking sector, as described in Figure 1.

All studies in this review show that workplace stress is a critical banking sector issue with potentially negative effects on workers' and organizations' psychological and physical health. Most of the studies showed increases in mental health problems in the sector which were closely related to stress at work. Authors have used a number of different parameters to investigate banking sector job stress: some (Silva and Barreto, 2010, 2012; Snorradóttir et al., 2013; Petarli et al., 2015; Valente et al., 2015) used the Demand Control (Karasek, 1979) and Effort-Reward Imbalance (ERI) (Siegrist, 1996) theoretical models. These studies showed a link between several undesirable mental and physical health outcomes and stress related to work. Silva and Barreto (Silva and Barreto, 2010, 2012) found a statistical association between adverse working conditions and minor psychiatric disorders (MPD) and a more than doubling of MPD where staff found themselves in conditions of maximum demand and minimum control. Snorradóttir et al.'s (2013) examination of psychological trauma among surviving bank employees in restructuring and downsizing processes showed that such environment factors as high job demand and low job control were fingered to a limited degree in perceptions of psychological distress, and that empowering leadership and family/friend support could at least partly mitigate the effect.

Petarli et al. (2015) saw social support as important and this is seen as best-known situational variable in occupational stress (Bakker and Demerouti, 2007): low social support made it more likely that a worker would be in the “high distress” quadrant (high-demand and low-control jobs). Where a quadrant intimated higher risk of occupational stress, variables associated with that quadrant were low levels of education, working in bank agencies, having worked more than 5 years at the bank, daily 6-h work shifts of, and—in particular—low social support. Valente et al. (2015) showed that working roles seen as high strain, low social support, high effort/low reward and high over-commitment correlated strongly with depressive symptoms both major and lesser.

Michailidis and Georgiou (2005) looked for factors contributing to occupational stress for employees in this sector, and identified the degree of occupational stress experienced by people in different groups. Evidence was presented educational levels, family support and drinking habits had an effect on the degree of stress experienced, while Ahmad and Singh (2011) assessed how far occupational stress and certain biographical variables in a sample of Indian bank employees influenced employees' perceived reactions to organizational change (OC). Organizational stressors such as responsibility for people, intrinsic impoverishment, low status and unprofitability were all shown to have causative influence on banking sector employees' perceived reactions toward OC. The only biographical variable found to be predictive of banking sector employees' reactions toward organizational change was “experience in the present position.”

Preshita and Pramod (2014) researched differences between male and female respondents and between those employed in private and in public sector banks in the nature of stress. Moderate to high stress levels were felt in both sectors, and the most powerful role stressor in either sector was shown to be role stagnation, with inter-role distance second and role erosion third.

Associations between occupational stress and job burnout were investigated (Mutsvunguma and Gwandure, 2011; Amigo et al., 2014; Li et al., 2015). Li et al. (2015) showed that occupational stress may play a part in job burnout but that it can be mediated by Psychological Capital, which Luthans et al. (2005) defined as “a positive psychological state that an individual performs in the process of growth and development.” It comprises four holders of psychological resource; self-efficacy; hope; optimism; and resilience. Banking employers could decrease burnout by increasing PsyCap. Amigo et al. (2014) showed that Spanish bank staff had high levels of burnout syndrome (BS) and that emotional exhaustion was the chief factor. There was a greater risk of burnout for those working in branch offices than for those in central services and a close correlation between burnout and interpersonal stress at work on a daily basis because of the commercial strategies the sector has used in recent years. A comparison by Mutsvunguma and Gwandure (2011) of levels of work stress, burnout and life satisfaction showed that bank employees who handled cash had higher levels of stress, depersonalization, emotional exhaustion and burnout than those who did not. Exposure of cash-handlers to unpredictable violence was shown to be a powerful factor in this differentiation (Harrison and Kinner, 2010).

There was analysis from some authors of how work-related stress impacted specific conditions (Seegers and van Elderen, 1996; Mughal et al., 2010; Oginni et al., 2013; Imam et al., 2014; Kan and Yu, 2016). Kan and Yu (2016) researched how occupational stress and work-family conflict affected depressive symptoms, and whether PsyCap could mediate those effects. A number of occupational stressors associated strongly with PsyCap, which mediated at least in part the associations of symptoms of depression with extrinsic effort and reward, suggesting that in PsyCap the banks may have a resource to mitigate their employees' symptoms of depression. Imam et al. (2014) investigated job stress as a mediator between gender discrimination in the workplace and levels of job satisfaction and employee motivation, finding that stress was a partial but vital mediator not in reducing but in strengthening gender discrimination in Pakistani banks. Oginni et al. (2013) examined how job stress affected staff turnover in Nigeria's banking sector. Job security was shown to be the Number One source of Nigerian bankers' job stress, with work materials second, organizational policies third and work pressure (which is in some ways a follow-up to the organizational policies) coming after those. Mughal et al. (2010) measured how far job stress affected work-life balance and found that organizational sources of stress did have an impact on work-life balance, so that stressors can be said to be directly proportional to work-life balance. Seegers and van Elderen's (1996) research into work-related stressors and their impact on psychological and physical well-being and absenteeism used the Michigan model as a theoretical framework (Caplan et al., 1975), and this threw up an interesting relationship between the investigated variables. Subjective stressors and stressors related to work did well as predictors of psychological strains and complaints, which may become health problems for the affected employee, but the model is inadequate as a way to research absenteeism which remained largely unexplained.

Other researchers looked closely at role stressors in the banking sector (Fernandes et al., 2012; Devi and Sharma, 2013). Devi and Sharma's (2013) research into the role stressors of frontline Indian bank employees showed significant differences among frontline bank employees, who could be divided into three categories: “overloaded employees,” “unclear employees,” and “underutilized employees.” Employees in each of these categories had profiles different from those in the other categories and the results reinforced the need to customize approaches to role stress management. Fernandes et al. (2012) questioned what impact health, environmental, and organizational factors had on organizational role stress (ORS), which was measured using the scale developed by Pareek (1983), from which can be derived indices of individuals' perceived role stress. The authors were able to find specific factors for stress reduction, with human support chief among them; relaxed health practices and vigorous health practices came next. This study increased the available evidence for the idea permanent cures for workplace stress may be found using environmental, health, and demographics in the workplace as explanatory variables. Human relations were said to have overwhelming significance in relieving stress, which may be symptomatic of a culture—like India's—in which community plays the major role in everything.

Some authors concentrated on organizational stress's role in development of specific symptoms. Makhbul et al. (2011) sought to identify the most significant among the ergonomic workstation variables influencing stress levels in Malaysia's Banking Supervision Department. 47.2% of changes in workplace stress were shown to be due to posture and health factors. Posture was significantly related to workplace stress outcomes. Mocci et al. (2001) looked at how different stressors such as social environment, task, and individual characteristics influenced asthenopia (visual discomfort) in computer users, to study how far psychological stressors might be at the root of asthenopeic complaints. Social support was found to be a predictor of visual complaints, as were self-esteem, work satisfaction, group conflict, and the underuse of skills. At least some complaints about visual health made by workers were probably indirect expressions of psychological discomfort related to working conditions.

Most studies agreed that social support could provide protection against occupational stress thus be important in reducing perceived stress levels. Social support was shown to tend to mitigate negative effect of stressors and to reduce the volume of stress reactions, and could be considered the best-established anti-occupational situational variable (Seegers and van Elderen, 1996; Fernandes et al., 2012; Petarli et al., 2015). It could also act as a visual complaint predictor of (Mocci et al., 2001). A lack of social support in the work environment had a statistically association with minor psychiatric disorders (Silva and Barreto, 2010, 2012; Valente et al., 2015). This variable's predictive ability in reducing occupational stress confirmed results seen in other studies (Leong et al., 1996; Urbanetto et al., 2011). Social integration, confidence in peers and the support of colleagues and superiors when performing tasks, could protect workers' health against work-related stress and its effects, and it is interesting that cortisol, the hormone released during stress, was found in increased amounts in women whose social support was low (Evolahti et al., 2006). This result strengthened the evidence for the protective effect of social support. Snorradóttir et al. (2013) presented contradictory findings, in that it found social support from friends and family to be a stress reducer for employees involved in organizational change, but found no such effect when the support was provided by coworkers or supervisors. This study did show, though, a correlation between coworker and supervisor support and empowering leadership. Other studies have also found effects of work-related support to be limited following downsizing (Lavoie-Tremblay et al., 2010). One reason could be that organizational change disrupts social bonds, especially soon afterwards when new social bonds have not had time to form (Shah, 2000); this study does offer support for that view, since employees going through organizational change were less likely than others to say they felt they had received good support from supervisors or coworkers. Support from friends and family, on the other hand, continued to be important for mental health when rapid and unpredicted organizational change loomed (House, 1980; LaRocco et al., 1980; Snorradóttir et al., 2013).

Several studies investigated what effect demographic characteristics had on workplace stress. Looking at age, Silva and Barreto (2012) found that being over 40 had a significant association with poor self-rated health; Kan and Yu (2016) measured a significantly higher level of depressive symptoms in bank employees over 40 than in those aged 30; Amigo et al. (2014) saw age's effect in Burnout Syndrome scores, with those over 55 showing less “emotional exhaustion” and less “cynicism” than those between 46 and 54, while under-35s saw themselves as more professionally efficient than any older group on the “efficacy” scale. Other authors, however, found no significant differences related to the age variable (Snorradóttir et al., 2013; Valente et al., 2015).

On years of service, Petarli et al. (2015) found that those who had worked in the bank for more than 5 years were more likely to fall in the “passive” (intermediate risk of stress) quadrant than those employed for <5 years; in Ahmad and Singh (2011) the only biographical variable to act as a predictor of employees' perceived reactions toward Organizational Change was “experience in the present position”; Amigo et al. (2014) reported that those with more than 30 years of service scored significantly lower on the burnout's cynicism scale than any of the shorter-service groups, whereas workers with <10 years of service had a higher efficacy score than any other group. Valente et al. (2015) found an association between total years worked and MDS (major depressive symptoms), resulting from the continuous updating of skills required in banking to keep up with new organization formats; researchers wondered whether this added extra stress, especially for older workers, who might feel threatened by such pressures (Giga and Hoel, 2003). On the other hand, Silva and Barreto (2010) found that the prevalence of minor psychiatric disorders showed little variation linked to duration of employment or job position.

Results with regard to the level of education variable conflicted with each other. Michailidis and Georgiou (2005) showed marked differences between bank employees with higher degree level qualifications, and those without formal qualifications. The former seemed most affected by home/work interaction and tended to take work problems and demands home, appearing to pursue a career to the detriment of home life. They were also most affected by the work variety, favoritism, delegation and conflicting task variables. The study also showed that, when those issues reached a high level, they tended to reduce the time employees had for themselves. On the other hand, Petarli et al. (2015) evaluated occupational stress on the demand-control model (Karasek et al., 1998) and showed that low education increased an employee's probability of being in the “passive” (intermediate risk of stress) quadrant. And, last, Kan and Yu (2016) detected no significant difference in bankers' symptoms of depression stemming from the education variable.

Several authors looked for gender differences. Snorradóttir et al. (2013) reported higher levels of psychological distress among women; Li et al. (2015) saw gender differences in mediation by Psychological Capital of the occupational stress/job burnout association, with PsyCap mediating the effects of two dimensions of occupational stress (extrinsic effort and reward) on two dimensions of job burnout (emotional exhaustion and depersonalization) for male staff while, for female bank employees, PsyCap partially mediated the effects of three dimensions of occupational stress (extrinsic effort, reward and over-commitment) on two dimensions of job burnout. Amigo et al. (2014) also found significant differences in emotional exhaustion and professional efficacy, with women scoring higher for emotional exhaustion and men scoring lower for professional efficacy. Fernandes et al. (2012) reported that females experienced more stress than males and attributed this to domestic pressures and increasing demands in the workplace. Other authors, however, reported no significant gender difference (Silva and Barreto, 2010, 2012; Preshita and Pramod, 2014; Valente et al., 2015; Kan and Yu, 2016).

Finally, some researchers researched which work characteristics were most associated with stress. Petarli et al. (2015) said that bank agency workers were more likely to belong to the “high distress” quadrant than workers in the administrative unit. A study by Valente et al. (2015) suggested that private bank or branch office work were most associated with ODS (other depressive symptoms). There was also a higher risk of employment instability and downsizing for those in the private bank sector which led to greater pressure at work, more stress and a higher rate of overload (Giga and Hoel, 2003). Preshita and Pramod (2014) also found higher total organizational role stress scores in private sector employees. Possible causes lie in the strict deadlines and lack of job security in private sector banking. Mutsvunguma and Gwandure (2011) examined how the psychological functioning of bank employees who handled cash and those who did not differed and found significant differences in work stress, emotional exhaustion, depersonalization and overall burnout. This study's findings corroborated existing research evidence to the effect that exposing employees to unpredictable and violent work environments could raise psychological distress levels and impair employee effectiveness (Harrison and Kinner, 2010). Amigo et al. (2014) split the study population into two groups: workers not working with the general public; branch office workers. There were significant differences between the two groups in all three burnout dimensions: emotional exhaustion, cynicism and professional efficacy. Branch office workers returned higher scores for emotional exhaustion and cynicism and lower scores for professional efficacy. Branch office workers were in daily contact with people with serious economic problems and were also required to sell their customers complicated financial products, and all of these things took away their control over aspects of their working lives and might explain their feelings of lower professional efficacy and greater emotional exhaustion and cynicism (Alarcon, 2011; Lee et al., 2011).

The relationship between work-related stress and mental disorders were confirmed also in other sectors, as described in a recent systematic meta-review of work-related risk factors for common mental health problems (Harvey et al., 2017). The Authors observed as certain types of work may increase the risk of some mental disorders, but the nature of the relationship is far from clear and is still a subject of debate. Nevertheless, the results of our review confirm, on the one hand, the initial assumption that psycho-social disorders of bank employees are increasing and, on the other hand, that—even though we could not identify specific clinical frameworks—banks are nowadays an occupational sector particularly at risk for work-related stress.

This review was conducted according to scientific criteria and explored the main stress-related aspects in a changing productive sector such as credit industry. This is a strength, but there are also some limitations, which may affect the results. First, this is not a systematic review, so it has not been designed to provide a complete and exhaustive summary of current literature relevant to the research question. Second, studies overviewed in this paper are all cross-sectional, none used objective parameters, and all were self-reporting. One may add to that the fact that only studies in which the keywords appeared in the abstract or title were included. Moreover, only MEDLINE® was explored to give this first description of work-related stress in the banking sector. All these aspects may have led to not including all the articles related to the investigated topic, so limiting the extent of knowledge of the effects and features of work-related stress in the banking sector. Future systematic reviews, including other databases as well, will be performed to deeply describe the state of the art of this phenomenon according to specific issues, such as risk factors or health outcomes.

Finally, the overview was restricted to studies published in the English language, so limiting the extent of knowledge of the effects and features of work-related stress in the banking sector.

In conclusion, occupational stress has clearly become a significant cause of ill health and is a serious risk factor for bank workers' psychological and social well-being. This literature review has demonstrated an increasing diffusion of adverse health outcomes from work-related stress in this sector.

There is a need for further studies to provide a better analysis of the relationship between work- related stress and health in the banking sector. It would be particularly interesting to carry out longitudinal studies to identify changes in the level and incidence of health problems and to map variations in economic, organizational, and social conditions. Future research should couple longitudinal designs with both objective and subjective measurements of stressors from a number of sources to increase understanding of organizational stress. Future research should also evaluate changes in the different groups of bank employees resulting from actions taken in organizations.

## Author contributions

GG, GA, AD, and NM conceived and designed the review; MP, CL, and JF-P performed the literature research; GG, NM, and AA-M analyzed the data; GG and NM wrote the paper.

### Conflict of interest statement

The authors declare that the research was conducted in the absence of any commercial or financial relationships that could be construed as a potential conflict of interest.
